# Arthroscopic and endoscopic techniques for iliopsoas release in THA are safe and effective: a systematic review of the literature

**DOI:** 10.1007/s00590-024-04042-1

**Published:** 2024-07-08

**Authors:** Riccardo Giai Via, Matteo Giachino, Ahmed Elzeiny, Andrea Donis, Simone De Vivo, Francesco Bosco, Alessandro Massè

**Affiliations:** 1 Department of Orthopaedic Surgery, Centro Traumatologico Ortopedico (CTO), Turin, Italy; 2 Department of Orthopaedics and Traumatology, University of Turin, Turin, Italy; 3 Department of Orthopaedics and Traumatology, Faculty of Medicine, Kafr El Sheikh University, Kafr El-Shaikh, Egypt; 4 Department of Precision Medicine in Medical, Surgical and Critical Care (Me.Pre.C.C.), University of Palermo, Palermo, Italy; 5 Department of Orthopaedics and Traumatology, G.F. Ingrassia Hospital Unit, ASP 6, Palermo, Italy; 6 Department of Orthopedic and Traumatology (DICHIRONS), University of Palermo, VIA DEL VESPRO, 129-90127 Palermo, Italy

**Keywords:** Iliopsoas impingement, Iliopsoas tenotomy, Hip arthroscopy, Endoscopic, Arthroscopic, Total hip arthroplasty, Total hip replacement

## Abstract

**Background:**

Hip replacement surgery is highly effective in relieving pain and improving mobility in patients with various hip conditions. However, some patients develop groin pain after surgery, often due to iliopsoas impingement (IPI), which can be challenging to diagnose. Conservative treatments are initially recommended, but when these are not effective, surgical options may be considered. This study aims to evaluate the clinical outcomes, success and failure rates, revision rates, and complications associated with arthroscopic and endoscopic surgery for IPI, thereby providing a comprehensive understanding of the effectiveness and risks of these surgical interventions.

**Materials and methods:**

A systematic review was conducted following Preferred Reporting Items for Systematic Reviews and Meta-Analyses (PRISMA) guidelines, including a thorough search of five main databases: PubMed, Scopus, Embase, Medline, and Cochrane. Eligible articles were meticulously evaluated according to predefined criteria for levels of evidence (LoE), with retrospective studies assessed using the Coleman Methodology Score (mCMS). This systematic review was registered in the International Prospective Registry of Systematic Reviews (PROSPERO).

**Results:**

Among the 16 included studies, 431 patients with 434 hips underwent either endoscopic or arthroscopic tenotomy. Both techniques showed favorable outcomes, with arthroscopic tenotomy demonstrating slightly higher success rates than endoscopic tenotomy. Common complications included mild pain and occasional infections, with recurrence observed in some cases. Both techniques offer direct visualization of prosthetic components and potential preservation of psoas function.

**Conclusions:**

Arthroscopic and endoscopic iliopsoas tenotomy are effective treatments for alleviating symptoms and improving hip function in patients with IPI post-total hip arthroplasty (THA).

**Level of evidence:**

IV.

## Introduction

Hip replacement surgery effectively treats problems such as degeneration, arthritis, and severe pain. The surgery relieves pain, improves mobility and function, and increases overall well-being for many patients. However, after surgery, some patients experience groin pain with a prevalence ranging from 0.4 to 18.3% [1, 2]. Among the potentially most underdiagnosed causes of inguinal pain is iliopsoas impingement (IPI), which, according to the literature, has a frequency of 4.4% [2–5]. The IPI may be due to several factors: acetabular cup fixation screws that are too long and penetrate the ileum, osteophytes, excess cement in the case of cemented acetabular cups, large femoral heads, or protruding cups [6–11]. In some patients, those with acetabular dysplasia, hypoplasia of the anterior wall creates a higher risk of impingement with the iliopsoas tendon: a prominent cup induces friction and irritation on the iliopsoas tendon itself, resulting in bursal effusion, tendinitis, and sometimes partial rupture [4, 12, 13]. Patients with this condition present with groin pain while climbing stairs, lying in bed, or getting out of a car. It may be perceived as a snapping or clanking sensation [4, 8]. The onset of pain varies from immediately after surgery to several years afterward. Clinically there is no specific test, although a painful resisted straight-leg raise test or pain with passive hyperextension are common findings [4, 14, 15]. The diagnosis of IPI is clinical, but an anteroposterior (AP) X-ray of the pelvis and a lateral X-ray of the hip provide a better assessment of cup placement [4, 5, 8]. Other imaging examinations such as ultrasound, computed tomography (CT) scan, or magnetic resonance imaging (MRI) with metal artifact reduction sequence (MARS) provide a more accurate diagnosis and allow better study of the positioning of prosthetic components that may cause impingement [16–18].

For this pathology, the first line of treatment is conservative with non-steroidal anti-inflammatory drugs (NSAIDs), targeted physiotherapeutic stretching exercises and possible ultrasound-guided infiltrative therapy at the level of the iliopsoas with local anesthetics, corticosteroids or even botulinum toxin [19, 20]. Resolution of symptoms with nonsurgical treatment is expected in about 47–50% of patients [5, 21]. Surgical treatment is proposed for patients who do not respond to conservative therapy and with significant symptomatology. In the past, the most used surgeries were open tenotomy of the iliopsoas for patients with acetabular prominence < 8 mm or revision of the acetabular component for patients with acetabular prominence > 8 mm [4, 5]. In any case, open-release surgery is a moderately invasive procedure and can be technically challenging, particularly in the presence of a previous hip approach. All this has led to the increased use of minimally invasive arthroscopic techniques for iliopsoas tenotomy for this type of patient.

Several methods are used for arthroscopic and endoscopic iliopsoas tenotomy: a tenotomy at the level of the lesser trochanter, with access to the distal insertion of the iliopsoas tendon, as described by Ilizaliturri in 2005 and by Williams in 2018 [22, 23]; or a transcapsular release at the level of the psoas notch, as described by Wettstein in 2006 [24].

This systematic review aims to comprehensively analyze the clinical outcomes, success rates, failure rates, revision rates, and associated complications of arthroscopic and endoscopic surgery for IPI in patients who have undergone hip replacement. By providing a detailed understanding of the effectiveness and risks of this surgical intervention, the findings will guide clinical decisions and enhance patient car.

## Materials and methods

### Search strategy and study screening

**Fig. 1:**
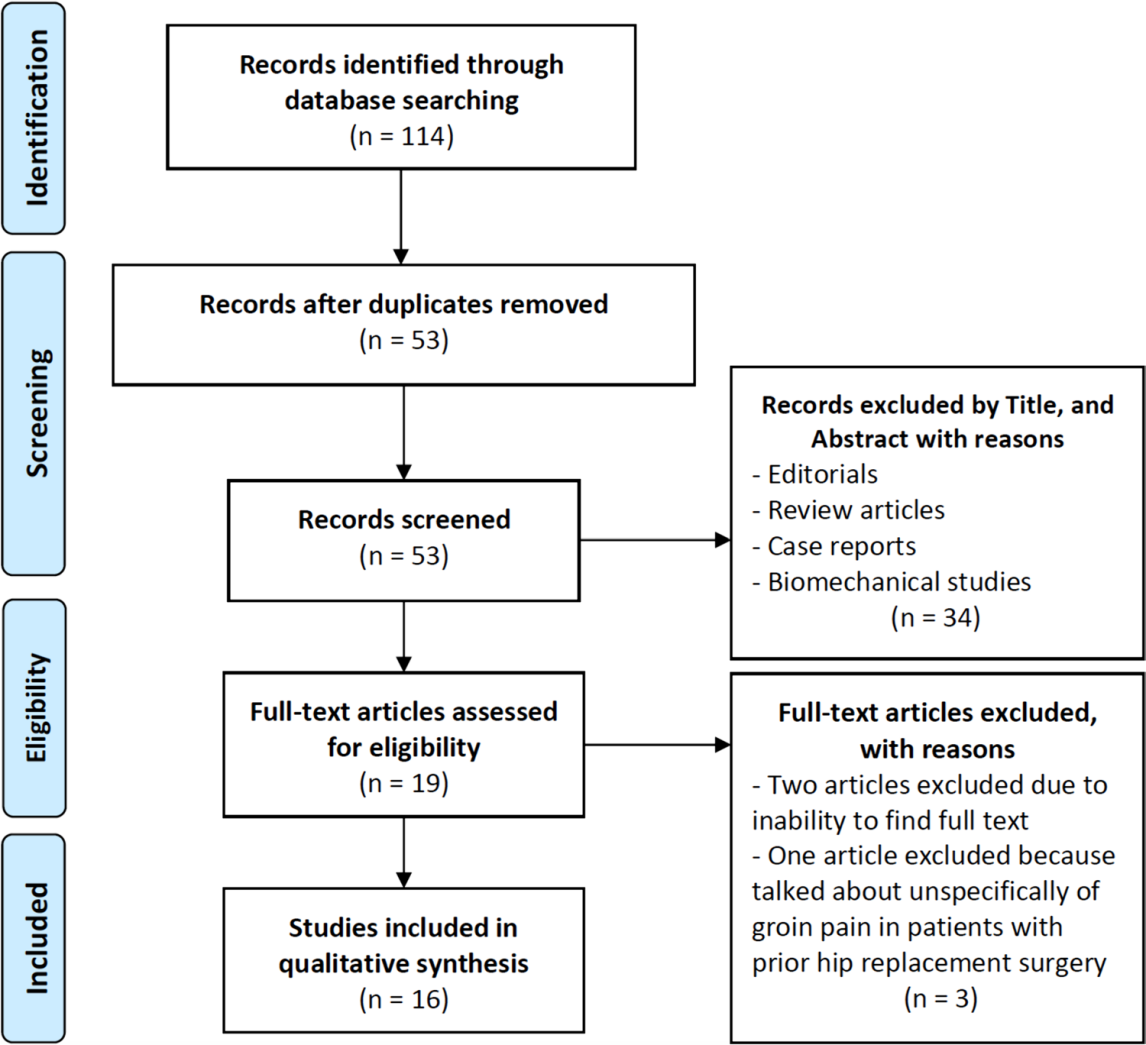
Preferred reporting items for systematic reviews and meta-analyses (PRISMA) flow diagram

A comprehensive and systematic literature search was conducted across five databases (PubMed, Scopus, Embase, Medline and Cochrane) utilizing the following MeSH terms: ((psoas) OR (iliopsoas)) AND ((tenotomy) OR (release)) AND ((endoscopy) OR (arthroscopy)) AND ((THR) OR (Total hip replac*) OR (THA) OR (total hip arthroplast*)). Three authors (RGV, AE and AD) independently conducted a literature search and evaluated studies to minimize mistakes. Uncertainties were resolved through consultation with a fourth author (MG). The search included 114 studies published from 2000 to February 2024. After removing duplicates, 53 studies were included. Following a review of the title and abstract of these studies, 34 studies were excluded, yielding 19 eligible studies. After full-text evaluation, 16 clinical studies met the qualitative analysis eligibility criteria. The included studies directly reported functional outcomes, time for resolution and improvement of symptoms, and differences in complication rates among patients undergoing arthroscopic iliopsoas tenotomy with a prior hip replacement surgery. This study adhered to the Preferred Reporting Items for Systematic Reviews and Meta-Analyses (PRISMA) guidelines [25] (Fig. 1).

### Inclusion and exclusion criteria

The inclusion criteria for the reviewed studies were articles about patients with prior hip replacement surgery undergoing arthroscopic surgery for iliopsoas tenotomy. These articles, published in English, involved human subjects, with publication dates falling between 2000 and February 2024 and a minimum mean follow-up of six months. Randomized controlled trials (RCTs) and prospective and retrospective studies with levels of evidence (LoE) between 1 and 4 were considered [26]. Biochemical and in vitro studies, case reports, editorials, book chapters, technical reports, pre-clinical studies, review articles and studies with LoE 5 were excluded for better-quality research.

### Methodological quality assessment

**Fig. 2:**
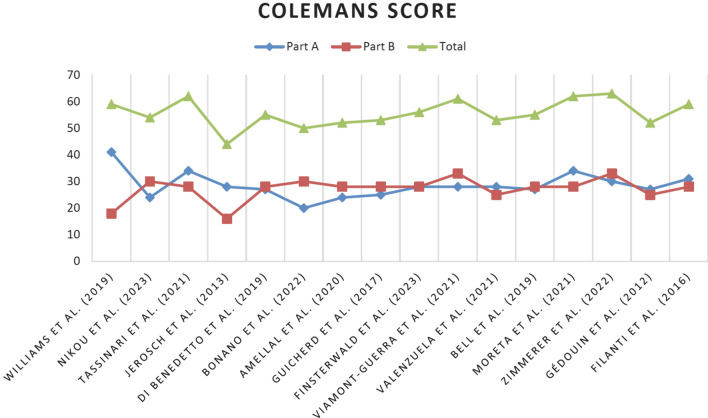
The coleman methodology score (mCMS), modified by ramponi et al. [27]

Each included article was evaluated according to the Oxford Centre for Evidence-Based Medicine 2011 LoE, which range from 1 to 5. The Coleman Methodology Score (mCMS), modified by Ramponi et al. [27], was used for retrospective studies (Fig. 2). Two authors (RGV, AE) employed this tool, with a third author (SDV) consulted for uncertainty resolution. All authors contributed substantially to the ideation and design of the study, data acquisition, manuscript writing, and final editing, and approved the final version of the article. This systematic review was registered in the International Registry of Systematic Reviews (PROSPERO), in March 2024 [28].

### Data extraction

Data extracted from the included articles were meticulously recorded in Excel spreadsheets by two independent authors (RGV and AE) and then subsequently unified. This included details such as the author and year of publication, study design, patient sample size, mean age, mean BMI, mean interval time between hip replacement surgery and symptoms onset, mean follow-up time, type of hip prosthesis, cup inclination and anteversion, type of anesthesia, rates of complications and recurrence, pre and post-operative subjective scores such as modified Harris Hip Score (mHSS), Visual Analogue Scale (VAS), Western Ontario and McMaster Universities Arthritis Index (WOMAC), Oxford Hip Score (OHS), International Hip Outcome Tool (iHOT33) or Copenhagen Hip and Groin Outcome Score (HAGOS). This facilitated organized data extraction and analysis, providing a comprehensive understanding of the study results.

### Data analysis

Statistical analysis used R software (version 4.1.3 from 2022), developed by the R Core Team in Vienna, Austria. Descriptive statistical methods were used for the data obtained from the included studies. Mean values were calculated for continuous variables, while variability was assessed through standard deviation (SD) or range (minimum–maximum) measures. Absolute numbers and frequency distributions were determined for categorical variables.

## Results

### Study characteristics

From the 114 studies initially retrieved, 16 studies met the inclusion criteria according to PRISMA flow chart [2, 23, 29–42]. Among these, seven studies focused on arthroscopic intracapsular iliopsoas tenotomy, seven on endoscopic extracapsular iliopsoas tenotomy, and two on both techniques. All patients underwent prior THR (primary or revision) or hip resurfacing and experienced post-operative iliopsoas tendinitis and failed conservative management that included physical therapy, injections, watchful waiting, or activity modification. Most studies were classified as level IV evidence and were predominantly retrospective (Table 1).

**Table 1:** Demographic data of included studies and patients

Authors (year of publication)	Study design (LOE)	Number of hips	L/R	No positive injection test	Age, years	M/F	BMI, kg/m2	FU, months	Symptomatic period, months	Time period from THA to IP tenotomy, years
		N°	N°/N°	N°	Mean ± SD (range)	N°/N°	Mean ± SD (range)	Mean ± SD (range)	Mean ± SD (range)	Mean ± SD (range)
Williams et al. (2019) [23]	Prospective (IV)	13	3/10	12	52.8 ± 13.7 (29.1–82.7)	2/11	30.6 ± 8.5	24 (6–84)	4 (2–24)	2.9 ± 2.2 (0.4–10.1)
Nikou et al. (2023) [29]	Retrospetive (IV)	12 (13)	8/5	NR	64.4 ± 15.1	4/8	26.6 ± 4.33	49.8 ± 25	9.3 ± 5.5 (0–52)	3.5 (1.75–8.3)
Tassinari et al. (2021) [30]	Retrospetive (III)	16	NR	All	57.8 ± 11.1	5/11	26.1 ± 3.9 (19–33)	27 ± 20.1 (6–48)	12.8 ± 20.1 (1–80)	3.4 (2–5)
Jerosch et al. (2013) [2]	Retrospetive (IV)	35	NR	All	58–82	NR	NR	43.2 (6–144)	NR	NR
Di Benedetto et al. (2019) [31]	Retrospetive (IV)	12	NR	NR	65 (47–82)	NR	NR	10 (3–12)	NR	NR
Bonano et al. (2022) [32]	Retrospetive (IV)	28	NR	20	69 (62.8–74.3)	8/20	30.4 (25.2–34.3)	7.6 (1–28)	NR	2.7 (1.5–6)
Amellal et al. (2020) [33]	Retrospetive (III)	19	NR	All	66 ± 11 (36–88)	9/10	28.1 ± 5.6 (21–43)	36 (6–66)	NR	4 (0.5–7.3)
Guicherd et al. (2017) [34]	Prospective (IV)	64	NR	48	56.3 (33–78)	24/40	26.0 (18.4–37.7)	8	40.3 (4–144)	NR
Finsterwald et al. (2023) [35]	Retrospetive (IV)	36	NR	31	62 ± 12 (27–83)	11/26	28.9 ± 4.3 (23.2–38.5)	24	NR	NR
Viamont-Guerra et al. (2021) [36]	Retrospetive (IV)	48(50)	NR	NR	60.8 ± 10.5 (35.5–80.3)	16/32	26.2 ± 4.8 (18.2–39.2)	31 ± 15 (12.3–71.5)	38.1 ± 29.9 (2–132)	NR
Valenzuela et al. (2021) [37]	Retrospetive (IV)	35	12/23	All	62 ± 10.33 (40–84)	15/20	28.74 ± 4.94 (20.3–41.1)	LT: 49.11 ± 20.46 AR: 42.42 ± 12.25	LT: 3.2 ± 5.42 AR: 4 ± 5.9	NR
Bell et al. (2019) [38]	Retrospetive (IV)	60	29/31	55	62.5 ± 9.7	24/36	29.6 ± 5.7	6	26.8	NR
Moreta et al. (2021) [39]	Retrospetive (III)	12	NR	All	59.1 (40–72)	6/6	27.2 (21.3–31.5)	45 (24–96)	12 (6–18)	1.7 (0.7–2.2)
Zimmerer et al. (2022) [40]	Retrospetive (IV)	20	8/12	All	59 ± 27.7 (52–78)	10/10	25.7 ± 5.5 (20.4–34.5)	7 ± 3.8 (2.6–11.7)	NR	6.3 ± 4.0 (1.7–15)
Gédouin et al. (2012) [41]	Retrospetive (IV)	10	NR	NR	58 (45–80)	5/5	NR	20 (12–60)	43 (14–72)	NR
Filanti et al. (2016) [42]	Retrospetive (IV)	11	NR	NR	57 (29–77)	6/5	NR	24	10.8 (5–15)	NR

NR = not reported; N° = number of evaluation cases; L = Left; R = Right; SD = standard deviation; FU = Follow-up; LT = lesser trochanter; AR = acetabular rim

### Demographics

This systematic review included 431 patients and 434 hips with IPI; 139 patients underwent arthroscopic intracapsular tenotomy, and 292 patients underwent endoscopic extracapsular tenotomy. The mean age of the patients varied between studies, as shown in Table 1. The sex distribution comprised 145 males and 240 females, with two studies omitting this information. Some studies reported the time from THA to tenotomy, and others mentioned the symptomatic period or both. Most studies reported body mass index (BMI) with mean values ranging from 25.7 to 30.6. All studies had a follow-up duration of at least six months (Table 1).

Diagnosis of IPI was established by clinical presentation, diagnostic injections, and imaging studies detecting acetabular prominence. Typical clinical presentation included groin pain during hip flexion as well as during the straight leg raise test. Radiographs included AP and latearal pelvis X-rays and pelvic CT scans to identify the prominence of acetabular components as a source of mechanical impingement. Pain provocation tests in the iliopsoas sheath were used in most studies for diagnostic confirmation (Table 1). Only two studies mentioned the American Society of Anesthesiologists (ASA) classification. In Williams et al., the mean was 1.8; however, in Bonano et al., the mean was 2 [23, 32].

Table 2 provides an overview of the characteristics of hip arthroplasty reported in the included studies. Most of the studies included were IPI after THA, except in five studies, which included ten patients with impingement after hip resurfacing. One study omitted data regarding the prosthesis [2]. Indications for surgery included failure of nonsurgical management, positive infiltration test, and signs of mechanical impingement on radiographs [2, 23, 32].

**Table 2:** Characteristics of hip arthroplasty of the included studies and patients

Authors (year of publication)	Characteristics of hip prosthesis	Cup frontal inclination (◦)	Cup anterior overhang (mm)	Cup anteversion (◦)
	N°	Mean ± SD (range)	Mean ± SD (range)	Mean ± SD (range)
Williams et al. (2019) [23]	2 Cemented THA, 11 Cementless THA	NR	NR	NR
Nikou et al. (2023) [29]	7 Cemented THA, 4 Cementless THA, 2 Hybrid THA	44.6 ± 6.9 (31–51)	8.71 ± 4.5 (1–14)	18.7 ± 6.8 (11–37)
Tassinari et al. (2021) [30]	16 Cementless THA	NR	13 ± 4.8 (5–20)	NR
Jerosch et al. (2013) [2]	35 THA	NR	NR	NR
Di Benedetto et al. (2019) [31]	11 THA, 1 Endoprosthesis, 1 Resurfacing	NR	NR	NR
Bonano et al. (2022) [32]	28 THA	45 (37.8–49)	7.0 (2.3–12)	30 (24.5–38)
Amellal et al. (2020) [33]	15 Primary THA, 1 Revision THA, 3 Resurfacing	45.7 ± 4.8	5.9 ± 2.8	12.3 ± 4.9
Guicherd et al. (2017) [34]	64 THA	44.8 (35–60)	NR	17.6 (0–38)
Finsterwald et al. (2023) [35]	21 Primary THA, 12 Revision THA, 3 Resurfacing	42.5 ± 7.2 (40–44.9)	NR	18.3 ± 9.5 (15.0–21.6)
Viamont-Guerra et al. (2021) [36]	42 Primary THA, 8 Revision THA	46.1 ± 7 (25–60)	6.9 ± 5.0 (0–20.5)	15.0 ± 8.6 (0–31.6)
Valenzuela et al. (2021) [37]	33 THA	NR	NR	NR
Bell et al. (2019) [38]	60 THA	44.1 ± 6.6	3.2 ± 6.7	13.4 ± 8.8
Moreta et al. (2021) [39]	12 THA	NR	7.25 (3–12)	NR
Zimmerer et al. (2022) [40]	20 Cementless THA	NR	5.5 ± 1.8 (2–8)	NR
Gédouin et al. (2012) [41]	9 THA, 1 Resurfacing	NR	NR	NR
Filanti et al. (2016) [42]	9 Primary THA, 2 Resurfacing	NR	NR	NR

NR = not reported; THA: total hip arthroplasty; N° = number of evaluation cases; SD = standard deviation; ° = degree

### Procedures performed

There were two main different techniques reported in the included studies as shown in Table 3.

**Table 3:** Surgical technique and postoperative therapy of patients following iliopsoas tenotomy

Authors (year of publication)	Anesthesia	Surgical technique	Post operative rehabilitation
Williams et al. (2019) [23]	Spinal	Endoscopic extraarticular tendon release (1–2 cm from LT) ± distal tendon stump (1 cm) removal	Full WB with crutches with hip extension/stretching exercises and hip flexion/strengthening exercises
Nikou et al. (2023) [29]	General	Arthroscopic tendon released at acetabular cup	Full WB with crutches for first days, physical therapy immediately
Tassinari et al. (2021) [30]	General	Arthroscopic transcapsular tenotomy until the muscular IP fibers	Full ROM and WB (with crutches) Capsular and iliopsoas stretching exercises
Jerosch et al. (2013) [2]	General	Arthroscopic tenotomy of the tendinous part	Patients according to their pain allowed to fully WB
Di Benedetto et al. (2019) [31]	NR	Arthroscopic tendon release at anterior rim of acetabular cup (one patient ossification excision)	No WB and assisted rehabilitation with passive ROM for 2 weeks Avoid active hip flexion with straight leg raise for 4 weeks
Bonano et al. (2022) [32]	NR	Endoscopic tenotomy at LT	Protected WB and no resisted hip flexion of 6 weeks
Viamont-Guerra et al. (2021) [33]	NR	Endoscopic IP tenotomy at the level between LT and the psoas valley on the acetabular rim	Full WB allowed as tolerated, cryotherapy started immediately with iliopsoas stretching, psoas strengthening started after 1 month
Amellal et al. (2020) [34]	General	Endoscopic tenotomy at LT	Full WB was immediately allowed Iliopsoas stretching, muscle strengthening
Guicherd et al. (2017) [35]	NR	Endoscopic tenotomy at LT: 57 hips Arthroscopic transcapsular tenotomy: 7 hips	Post–operative physiotherapy prescribed for 44% of patients
Finsterwald et al. (2023) [36]	NR	Endoscopic tenotomy at LT	Full WB with or without the use of crutches Avoid repetitive and resisted hip flexion first 6 weeks
Valenzuela et al. (2021) [37]	NR	Endoscopic tenotomy at LT: 21 hips Arthroscopic tenotomy at acetabulum rim: 14 hips	Full WB as tolerated, physiotherapy first 2 weeks keep hip flexion under 90, hip flexion and stretching exercises after 6 weeks
Bell et al. (2019) [38]	NR	Endoscopic tenotomy at LT	Protected WB for 2 weeks and resisted hip flexion for 6 weeks
Moreta et al. (2021) [39]	NR	Arthroscopic tenotomy at the edge of the acetabular	Full ROM and WB allowed with stretching exercises of iliopsoas Strengthening hip flexion exercises delayed for 3 months
Zimmerer et al. (2022) [40]	General	Arthroscopic transcapsular tenotomy	First 2 weeks, hip flexion and WB with crutches After 2 weeks strengthening abductors and core muscles
Gédouin et al. (2012) [41]	NR	Endoscopic extra–articular tenotomy	Full WB allowed, with two canes for support for 3 weeks
Filanti et al. (2016) [42]	NR	Arthroscopic transcapsular tenotomy	Full ROM allowed immediately with capsular and iliopsoas stretching Partial WB using crutches for 2 weeks

NR = not reported; WB = weight bearing; ROM = range of motion; LT = lesser trochanter; IP = iliopsoas; WB = weight-bearing; ROM = range of motion

### Endoscopic iliopsoas tenotomy

Seven studies used an endoscopic technique initially described by Ilizaliturri et al. involving extra-articular release against the lesser trochanter with the patient in the supine position [22]. In this technique an inferior visual portal and a superior working portal were used with the hip in flexion and external rotation. An extra-articular approach was used to limit the potential for damage to the prosthetic components. Variations in technique were noted, such as Williams et al**.** described release of psoas tendon 1–2 cm from LT with or without distal tendon stump (1 cm) removal [23].

### Arthroscopic iliopsoas tenotomy

Seven studies used a transcapsular arthroscopic technique with the patient supine and hip flexed at 30° to relax the anterior capsule. Anterolateral (AL) and mid-anterior (MA) portals were created for transcapsular tenotomy. After an extensive debridement, a transcapsular tenotomy was performed according to the Wettstein technique [24] with a radiofrequency probe while keeping the musculature intact. Di Benedetto et al. mentioned one hip endoprosthesis patient with significant periprosthetic ossification that underwent excision [31].

Guicherd et al. and Valenzuela et al. mentioned using both techniques (endoscopic and arthroscopic release). Seven studies stated the type of anesthesia; five were general, and one was spinal [2, 23, 29, 30, 34, 40]. Only 1 study reported the duration of surgery time, which was 23.7 ± 10.6, ranging from 6 to 55 min [34].

### Outcome data

The studies included in this systematic review used a variety of subjective outcome scores, as reported in Table 4. A successful outcome was defined as complete pain relief or lack of significant residual pain as assessed by the authors [2, 23, 29–42] at the final follow-up. In the case of arthroscopy, 89.4% of patients experienced favorable outcomes, while for endoscopic tenotomy, the successful outcome rate was 81.3%. For the two studies that used both arthroscopic and endoscopic techniques, one of them didn’t categorize the results of the two techniques [35]. Whereas the study from Valenzuela et al. mentioned that both modified Harris hip score (mHHS) and Non-Arthritic Hip Score (NAHS) showed superiority in the endoscopic group, though with no statistical significance (*p* = 0.06) [37]. Only one study, conducted by Williams et al., provided the timeframe for improvement, which ranged from 6 to 12 weeks [23].

**Table 4:** Summary of post operative outcomes, complications, recurrences and revisions following arthroscopic iliopsoas tenotomy

Authors (year of publication)	Pre op outcome; mean (range)	Post op outcome; mean (range)	Recurrence	Revision	Complications	Surgical technique	Successful Outcome
			N°	N°	N°		N°/ N° (%)
Williams et al. (2019) [23]	FABER test: all positive	FABER test: N°:9 negative, N°:2 positive, N°:2 unable	1	1	5 (mild pain)	Endoscopic	8/13 (62%)
Nikou et al. (2023) [29]	iHOT-12a*: 24.9 ± 13.8 HAGOS symptoms*: 38.2 ± 17.6 HAGOS-pain*: 36 ± 18.3 HAGOS-sport*: 14.1 ± 10.4 HAGOS-daily activity*: 31 ± 23.5 HAGOS-physical activity*: 21.8 ± 22.5 HAGOS- quality of life*: 24 ± 10.7 EQ-5D*: 0.339 ± 0.368 EQ-VAS*: 57.9 ± 15.9	iHOT-12a*: 39.5 ± 19.6 HAGOS symptoms*: 54.5 ± 33.1 HAGOS-pain*: 53 ± 30.3 HAGOS-sport*: 35.1 ± 22.1 HAGOS-daily activity*: 47.5 ± 28.6 HAGOS-physical activity*: 24 ± 21.9 HAGOS- quality of life*: 35 ± 20.9 EQ-5D*: 0.127 ± 0.385 EQ-VAS*: 58 ± 22.4	0	0	2 (mild pain)	Arthroscopic	10/12 (83%)
Tassinari et al. (2021) [30]	WOMAC*: 36.1 ± 8.6 (28–44)	WOMAC*: 83.4 ± 9.5	1	1	2 (mild pain)	Arthroscopic	13/16 (81.3%)
Jerosch et al. (2013) [2]	Presence of pain	Elimination of pain: 33/35	0	0	2 (mild pain)	Arthroscopic	33/35 (94%)
Di Benedetto et al. (2019) [31]	HHS*: 66.8 (48.9–81.8) MRC scale*: 3.6 (3–4) Hip flexion*: 95° (80°-100°) VAS*: 3.6 (2–6)	HHS*: 85 (80–95) MRC scale*: 4.7 (3–5) Hip flexion*: 105° (90°-120°) VAS*: 1 (0–3)	0	0	0	Arthroscopic	100%
Bonano et al. (2022) [32]	mHHS*: 57 (43–60)	mHHS*: 75 (66–92) VAS*: 3 (0.8–5) iHOT-12*: 71 (48–80)	1	1	2 (infection)	Endoscopic	17/28 (71%)
Amellal et al. (2020) [33]	VAS*: 6.15 ± 1.16 (4–8) OHS*: 25.2 ± 6.4	VAS*: 2.4 ± 2.6 (0–7) OHS*: 38.02 ± 12	4	0	3 (Hematoma)	Endoscopic	15/19 (79%)
Guicherd et al. (2017) [34]	OHS*: 21.8 Strength differential (MRC)*: − 1.7	OHS*: 40 Strength differential (MRC)*: − 0.7	0	0	1 (dislocation) 1 (hematoma)	Endoscopic:57 hips; Arthroscopic: 7 hips	94%
Finsterwald et al. (2023) [35]	mHHS*: 59.0 ± 19.5 (18.7—94.6) VAS*: 5.7 ± 1.7 (2.9–9) SANE-hip*: 54.3 ± 22.6 (1–91)	mHHS*: 82.9 ± 11.9 (61.6—100) VAS*: 2.1 ± 2.1 (0–7) SANE-hip*: 75.7 ± 17.1 (40–100)	0	0	1 (pain)	Endoscopic	97.2%
Viamont-Guerra et al. (2021) [36]	mHHS*: 57.7: ± 11.5 (31.9–81.4) OHS*: 29.4 ± 11 (4.-50)	mHHS*: 83.2 ± 16.9 (0–100.1) OHS*: 50.5 ± 8.4 (28.-60)	0	0	0	Endoscopic	40/50 (86.9%)
Valenzuela et al. (2021) [37]	VAS LT*: 5.32 ± 1.06 AR*: 5.75 ± 1.29	VAS LT*: 51.75 ± 1.77 AR*: 2.62 ± 2.22 mHHS LT*: 88.98 ± 10.29 AR*: 81.05 ± 12.44 NASH LT*: 85.99 ± 11.11 AR*: 78.85 ± 10.10	0	0	0	Endoscopic: 21 hips; Arthroscopic: 14 hips	(70.58%)
Bell et al. (2019) [38]	HOS(ADL)*: 57.5 ± 18.8 (10.9–89.3) HOS sports*: 37.3 ± 24 (0–83.3)	HOS(ADL)*: 71.6 ± 26.1 (14.1–100) HOS sports*: 58.1 ± 33.2 (0–100)	4	0	1 (asepting loosening) 1 (post-operative fall) 1 (hematoma) 1 (CRPS)	Endoscopic	56/60 (93.3%)
Moreta et al. (2021) [39]	VAS*: 6.2 (4–8) HHS*: 58.8 (37–76)	VAS*: 1.08 (4–8) HHS*: 86.1 (59–98) MRC scale*: 4.58 (4–5)	0	0	1 (mild pain)	Arthroscopic	11/12 (91.7%)
Zimmerer et al. (2022) [40]	mHHS*: 31.2 ± 9.8 (17.6–47.3) VAS*: 8.5 ± 1.2 (7–10) UCLA Score*: 4.0 ± 2.7 (0–7)	mHHS*: 82.0 ± 9.8 (46.2–100) VAS*: 2.5 ± 1.8 (0–6) UCLA Score*: 6.5 ± 1.8 (3–9)	0	0	2 (mild pain)	Arthroscopic	18/20 (90%)
Gédouin et al. (2012) [41]	PMA*: 13.1 (11–15) WOMAC*: 34 (24–46)	PMA*: 16.9 (15–18) WOMAC*: 84 (60–95)	2	0	0	Endoscopic	8/10 (80%)
Filanti et al. (2016) [42]	HHS score*: 46.4 (32–56) MRC scale*: 3.27 (3–4)	HHS score*: 83.3 (61–91) MRC scale*: 4.45	1	0	0	Arthroscopic	10/11 (86%)

N° = number of evaluation cases; % = percentage; CRPS = complex regional pain syndrome; mHHS = modified Harris Hip Score; WOMAC = Western Ontario and McMaster Universities Osteoarthritis index; iHOT-12 = International Hip Outcome Tool; HAGOS = Copenhagen Hip and Groin Outcome Score; EQ-5D = EuroQoL-5 Dimension Questionnaire; VAS = visual analogue scale; SANE-hip = Single Assessment Numeric Evaluation; NAHS = Non-Arthritic Hip Score; HOS(ADL) = Hip Outcome Score (activities of daily living); UCLA = Activity Score University of California Los Angeles; PMA = Postel M, Merle d'Aubigné; MRC = Medical Research Council; OHS = Oxford Hip Score; * = data expressed as mean ± SD (range)

Regarding the recovery of hip flexion strength after iliopsoas tenotomy, several qualitative methods have been used [30, 31, 35, 39]. Specifically, the MRC (Medical Research Council) score has been used in several studies [30, 31, 35, 39], showing good results, especially at six months follow-up. These results were comparable to the contralateral healthy side after surgery and showed statistically significant improvement over preoperative scores when applicable. Other studies have found improvements in flexion strength after surgery simply through clinical examinations at follow-up visits [32, 37]. Only one study, conducted by Finsterwald [36], used a quantitative method to analyze iliopsoas strength recovery using a dynamometer. This study [36] evaluated the recovery of strength in hip flexion both in supine position with the lower limb extended and in sitting position. The results demonstrated a reduction in flexion strength only in the sitting position, while no reduction was observed in the supine position.

### Complications

Post-operative complications, such as mild pain, were noted in the studies included in the systematic review, as reported in Table 4.

Bonano et al. documented two cases of post-operative infection that occurred at 7 and 10 months after surgery, necessitating a two-stage revision THA. One of these patients had previously undergone revision surgery for aseptic mobilization of the femoral stem one year before undergoing endoscopic psoas tenotomy. Notably, infections were not found to be related to the endoscopic procedure. In addition, recurrence occurred in two patients in the series: one underwent open tenotomy of the psoas due to persistent impingement. In contrast, the other patient showed no improvement in the mHHS. The authors suggested that both patients experienced relief from a preoperative steroid provocation test, and complete tendon release at the lesser trochanter level was confirmed in both cases, in addition to cup design/position, muscle tension/unbalance, posture or capsular tethering may have contributed to the recurrence. Specifically, the cup prominence in both cases was less than 8 mm, associated with less improvement in mHHS [32]. Guicherd et al. reported a case of anterior dislocation probably attributable to transcapsular tenotomy, along with a case of compressive hematoma involving the peroneal nerve, both promptly resolved by surgical drainage [35]. Amellal et al. observed three post-operative hematomas, all resolved spontaneously [34]. Nevertheless, Bell et al. reported four patients whose pain persisted after tenotomy. Among them, one patient had a recurrence of pain following a post-operative fall, another had aseptic loosening, and one patient developed a complex regional pain syndrome (CRPS). [38]. Gédouin et al. reported recurrence in two patients unresolved by infiltration, one of whom had periprosthetic ossification [41]. In addition, Filanti et al. documented one case of recurrence and post-operative dissatisfaction. [42]

## Discussion

The most important finding of this systematic review is that arthroscopic and endoscopic iliopsoas tenotomy in patients with THA may be an effective surgical procedure for treating symptoms and improving hip flexor weakness and functional outcomes. Two main techniques allow tenotomy of the iliopsoas: extracapsular (endoscopic) [22, 23] or transcapsular (arthroscopic) [24].

The studies analyzed in this systematic review show excellent outcomes for arthroscopic and endoscopic treatment. Of the seven studies analyzed in which patients were treated with extracapsular endoscopic technique, the average percentage of favorable outcomes was 81.3% (62–97.2%). Out of the seven studies that used transcapsular arthroscopic treatment, the average favorable outcome rate was 89.4% (ranging from 81 to 100%).

**Fig. 3:**
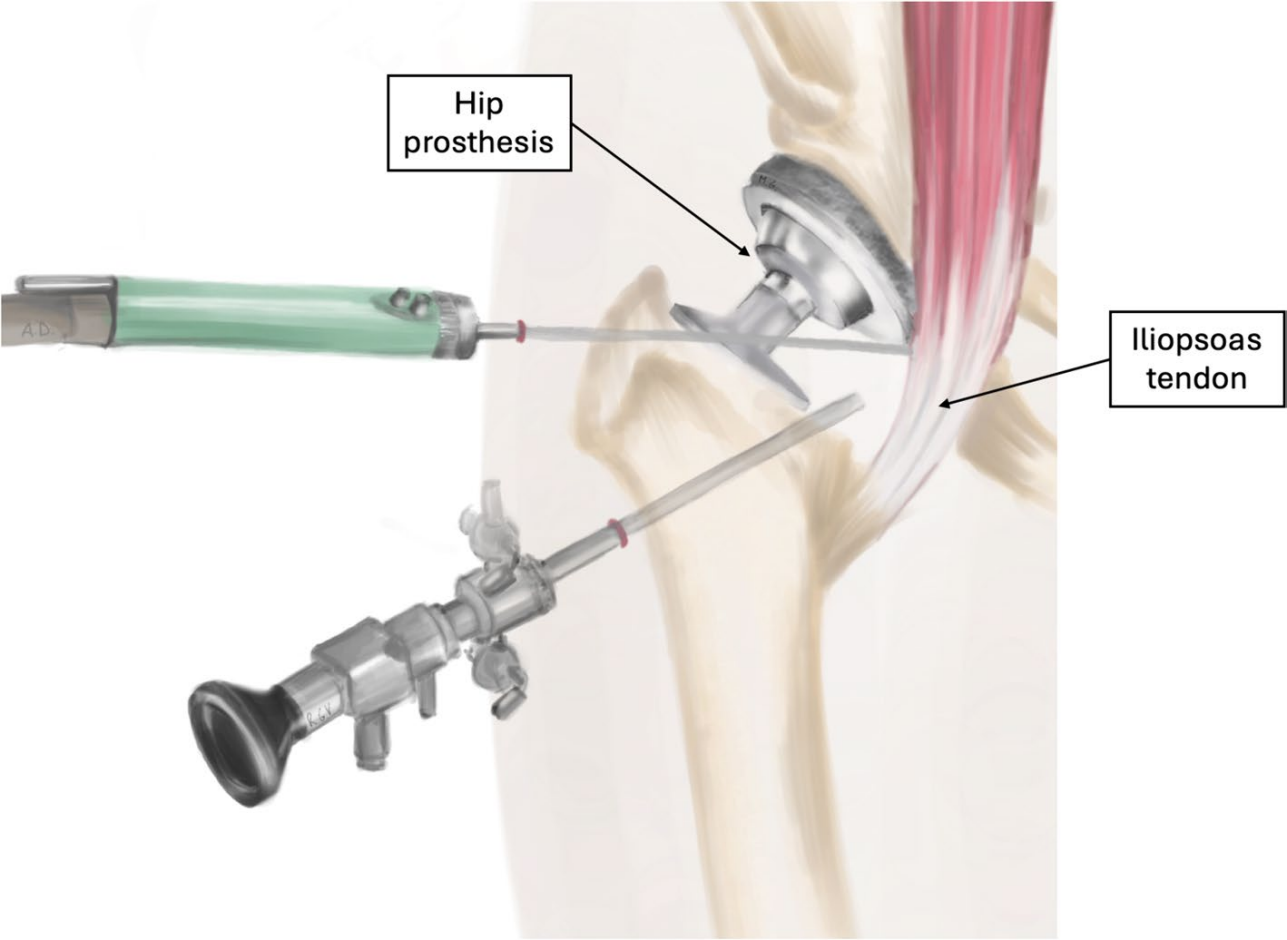
Illustration of the arthroscopic transcapsular technique for iliopsoas release surgery

The transcapsular arthroscopic technique (Fig. 3) has the advantage of directly visualizing the prosthetic bearing surface and anterior margin of the cup irritating the psoas tendon; thus, also having diagnostic value [35], it also allows the theoretical advantage of preserving greater function of the psoas since much of the muscle is at the intra articular level [29].

The endoscopic technique may be more accessible since it allows more direct identification of the iliopsoas tendon and reduces the risk of damaging the friction couple [35].

The main indication to recur to psoas release rather than directly to cup revision has usually been recognized as 8 mm maximum cup protrusion [4, 5]; it is interesting to observe that in the study of Tassinari et al., excellent results were recorded from arthroscopic release having an average cup protrusion of about 13 mm [30]. This is a significant finding because it allows this pathology to be approached even in cases with cup protrusion greater than 8 mm. It avoids direct recourse to cup revision, a more complex, invasive procedure burdened by a higher rate of complications.

None of the two studies [35, 37] that compared arthroscopic versus endoscopic treatment found statistically significant differences between one technique and the other, deferring the choice of technique preference to the surgeon. Guicherd et al. recommend giving an intracapsular look in case of diagnostic doubts in the case of iliopsoas tenotomy with an extracapsular endoscopic technique; in fact, they report instances of psoitis secondary to metallosis undiagnosed before surgery [35].

Concerning the recovery of hip flexion strength, Tassinari et al. reported superior outcomes in patients undergoing arthroscopic release [30]. Their findings indicated that this technique preserves a larger portion of the muscle belly, with 47% preserved in transcapsular tenotomy compared to 40% in endoscopic tenotomy [30]. Conversely, Valenzuela et al. observed an overall improvement in strength but did not find significant differences between the arthroscopic and endoscopic surgery groups [37]. In contrast, Guicherd et al. reported better recovery of hip flexion strength in patients who underwent physiotherapy [35].

Several studies report favorable outcomes, with no observed loss of strength following arthroscopic iliopsoas release, even in patients with painful internally snapping hips, whether associated with chondrolabral lesions or femoroacetabular impingement (FAI), in both general patients and athletes [43–45]. While most studies [30, 31, 35, 39] provide qualitative assessments, quantitative analysis using a dynamometer has been proposed by Finsterwald et al. [36]. This study [36] demonstrated that arthroscopic release of the iliopsoas was associated with a reduction in muscle volume and a decrease in hip flexion strength while seated. Nonetheless, the positive outcomes in hip flexion strength recovery reported in the literature may be attributed to findings by Márquez Arabia [46], which demonstrated via MRI that approximately six months post-surgery, a repair and reconstitution process of the iliopsoas tendon tissue occurs.

Regardless of the technique, full weight-bearing and free ROM are usually granted after surgery to reduce patient immobilization. These are typically associated with stretching and strengthening exercises of the iliopsoas to avoid adhesions and recover flexion strength limitation [36, 42].

Both arthroscopic and endoscopic techniques are not without risks; Guicherd et al. found anterior dislocation of the prosthesis due to excessive capsular debridement and a hematoma with compression and neuropraxia of the femoral nerve completely resolved after the hematoma was drained [35].

Overall, in the studies analyzed, except for the case of dislocation, no complications were reported, and the clinical outcomes were excellent for endoscopic and arthroscopic treatment. These techniques are, therefore, shown to be viable, safe, and reproducible for the management of IPI in patients with THA with a low grade of bias, as demonstrated by Coleman scores.

This systematic review is not without limitations, which must therefore be emphasized. First, most of the studies are retrospective. Second, there is no homogeneity of groups, patient selection, and post-operative program, nor follow-up. Third, not all studies performed an injection test before indication for surgery, a significant test in our opinion. Fourth, these studies are subject to various sources of bias in data collection and reporting, participant selection, and unblinded outcomes assessment that could affect the validity and reliability of the study conclusions. Five, a wide variety of follow-ups with 5.5–144 months were reported in the different studies. A more homogeneous and standardized clinical follow-up could improve the data’s validity. Therefore, it is essential to interpret the results cautiously and consider further research to confirm the results obtained in this systematic review.

Nevertheless, the findings presented in this systematic review lay the groundwork for formulating hypotheses in forthcoming high-caliber studies, as arthroscopic and endoscopic iliopsoas release are proving to be a reliable and productive approach for reducing pain, improving hip flexor function, and avoiding the need for cup revision in patients with symptomatic IPI after THA.

## Conclusions

This systematic review demonstrated that arthroscopic and endoscopic iliopsoas tenotomy effectively treat symptoms and improve hip function in patients with IPI after THA. Arthroscopic techniques allow direct visualization of prosthetic components and potential preservation of psoas function, while endoscopic techniques may be more straightforward. Both techniques show promise as alternatives to cup revision surgery. Further research with large-scale prospective randomized studies with carefully selected control groups and standardized protocols is needed to confirm these findings and optimize outcomes.

## Data Availability

Dataset analyzed in this study is available from the corresponding author on reasonable request.
